# Natural History of a Disease: Patent Ductus Arteriosus Diagnosed on an Elderly Woman

**DOI:** 10.7759/cureus.49519

**Published:** 2023-11-27

**Authors:** Georgia Rubiane Souza, Augusto Barreto do Amaral, Sarah Busch, Felipe Villa Martignoni

**Affiliations:** 1 Family Medicine, Complexo do Hospital de Clínicas da UFPR, Curitiba, BRA; 2 Internal Medicine, Central Montana Medical Center, Lewistown, USA; 3 Internal Medicine, University of Washington School of Medicine WWAMI, Lewistown, USA

**Keywords:** echocardiography, congenital heart disease, elderly, heart failure, patent ductus arteriosus, pda

## Abstract

Patent ductus arteriosus (PDA) is a rare finding in adults. The ductus arteriosus (DA) is responsible for shunting blood from the pulmonary artery into the aorta bypassing the lungs in fetal life (the placenta is responsible for fetal oxygenated blood). Its closure happens after birth, once fetal circulation transitions to normal postnatal circulation and blood oxygenates in the lungs. If the DA does not close, the PDA may continue to shunt blood from the systemic (higher pressure) to the pulmonary (lower pressure) circulation causing remodeling of the left ventricle and eventually heart failure. A PDA is suspected when there is a systolic/diastolic murmur in the left sternal border; a transthoracic or transesophageal echocardiogram may visualize the shunt and measure the systemic/pulmonary shunt ratio. We described a case of an 84-year-old elderly woman who presented with an acute heart failure exacerbation as the first symptom of PDA and was found to have left ventricular hypertrophy, right ventricular hypertrophy, and pulmonary hypertension as the initial presentation.

## Introduction

The prevalence of patent ductus arteriosus (PDA) is approximately 0.05%, making it a rare condition but the third most common congenital heart disease in adults [1]. Recognizing and treating this condition in the elderly is a challenge [2]. Once diagnosed, the prognosis is established and the benefit from intervention is to be determined by invasive measures.

## Case presentation

An 84-year-old woman was admitted to the emergency room (ER) with shortness of breath (SOB) on minimal exertion, orthopnea, and bilateral leg swelling that progressively worsened over the last three months. She endorsed no comorbidities and no medication use, but reported having a murmur during pregnancies (without any previous investigation). She has not been seen by a healthcare provider in the past 40 years.

Physical examination reveals a height of 167 cm, weight of 71 kg, and BMI of 25.4. Heart rate (HR) was 77 beats/min, blood pressure (BP) 137/69 mmHg, and O_^2^_ saturation in room air 84%. The neck has no lymphadenopathy or carotid bruit, but it has a pulsatile jugular venous pulse present at a 45-degree angle. Chest examination showed a 4+ systolic and diastolic harsh murmur in the second left parasternal space, regular heart rhythm, and normal rate. Breath sounds showed crackles bilaterally on the lower half of the thorax. Abdominal examination showed a normal liver size, no ascites, and non-tender to palpation. Legs had 3+ pitting edema bilateral; there was no cyanosis in extremities.

**Figure 1:**
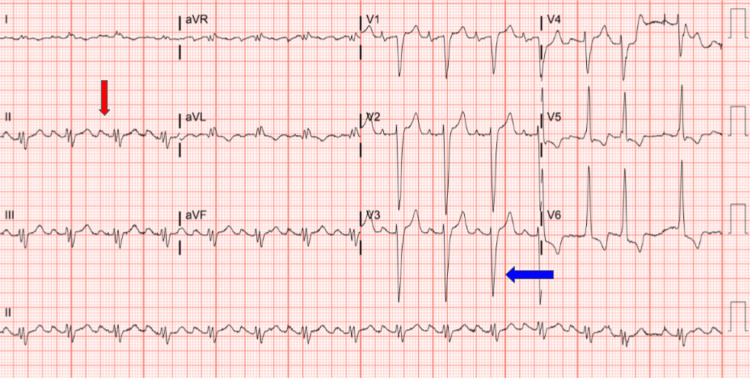
12 lead ECG showing sinus rhythm with left ventricular hypertrophy (indicated by the blue arrow) and bilateral atrial enlargement (indicated by the red arrow).

**Figure 2:**
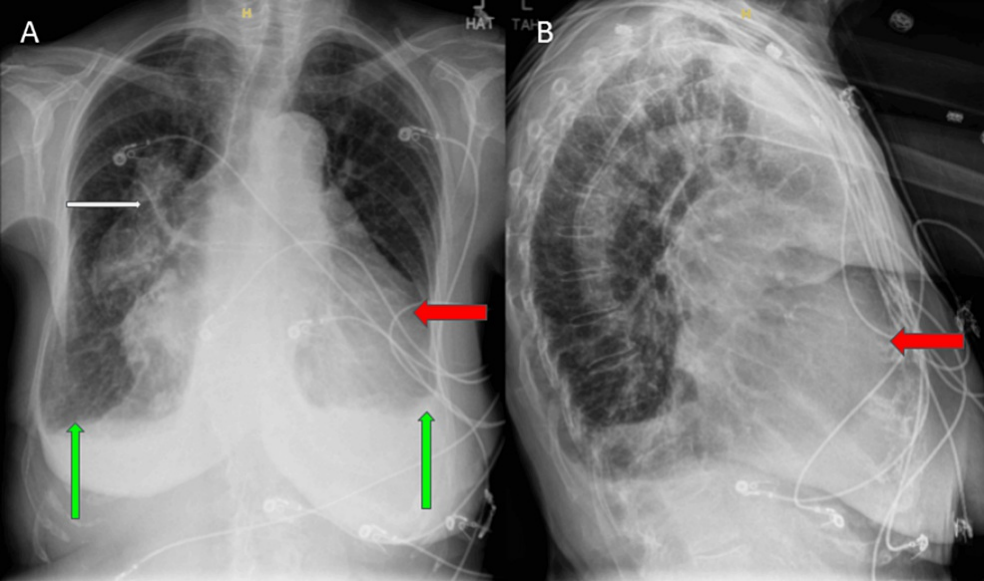
A: Chest X-ray showing globular cardiomegaly suggesting multi-chamber enlargement (indicated by the red arrow), interstitial pulmonary edema (indicated by the white arrow), and small bilateral pleural effusions (indicated by the green arrows). B: Chest X-ray showing cardiomegaly (indicated by the red arrow).

Laboratory biochemical analyses showed sodium of 139 mmol/L, potassium 4.4 mmol/L, chloride 101 mmol/L, CO_2_ 24 mmol/L, creatinine 0.89 mg/dL, blood urea nitrogen (BUN) 19 mg/dL, thyroid-stimulating hormone (TSH) 5.8 mg/dL, and N-terminal pro-brain natriuretic peptide (NT-proBNP) 8,756 pg/mL. The electrocardiogram (EKG) showed a normal sinus rhythm with signs of left atrial enlargement (LAE), right atrial enlargement (RAE), and left ventricular hypertrophy (LVH) with left axis deviation (Figure 1). Chest radiography revealed globular cardiomegaly suggesting multi-chamber enlargement, interstitial pulmonary edema, and small bilateral pleural effusions (Figure 2). The patient refused to do a computerized tomography (CT) of the chest.

**Figure 3:**
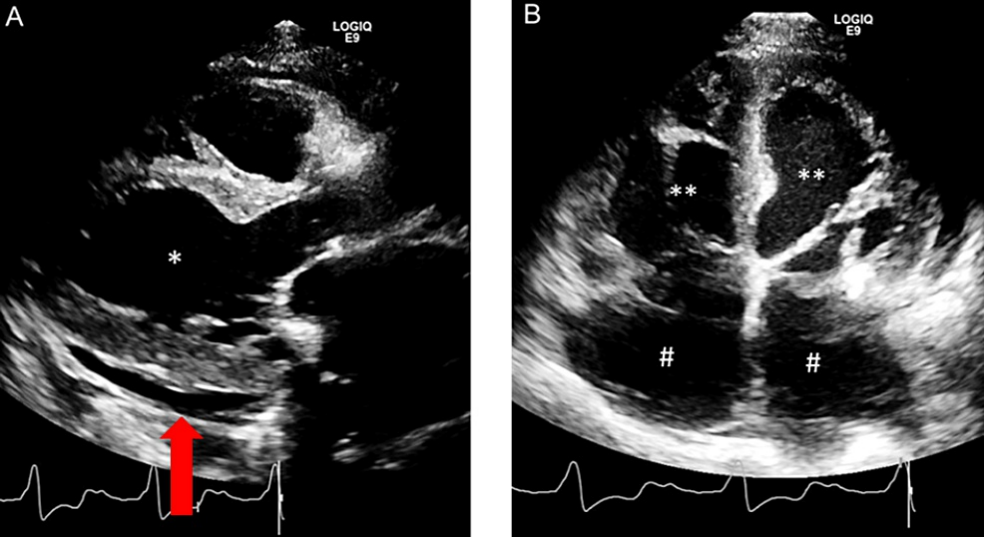
A: Parasternal long axis showing left ventricular concentric (*) and eccentric hypertrophy (*) and mild pericardial effusion (indicated by the red arrow). B: Four-chamber views showing left and right ventricular concentric (**) and eccentric hypertrophy (**), bilateral atrial enlargements (#), and mild pericardial effusion.

**Figure 4:**
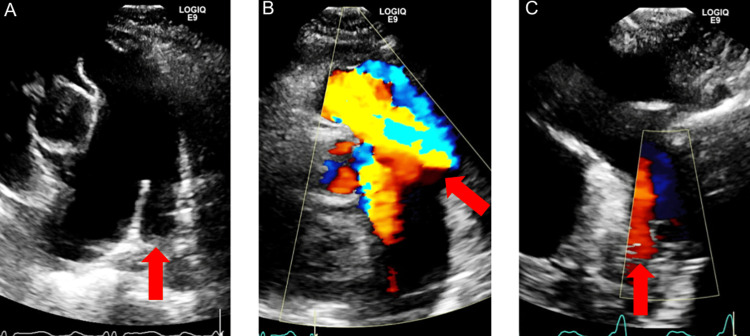
A: Parasternal short-axis view of the pulmonary artery showing a PDA (indicated by the red arrow). B: Arrow shows the flow into the pulmonary artery from the aorta. C: Suprasternal view showing color Doppler of the aortic arch and descending aorta with diastolic flow reversal (indicated by the red arrow).

The patient was admitted to the hospital for heart failure exacerbation and received intravenous furosemide. After diuresis, she became euvolemic within 48 hours of hospitalization and was not experiencing orthopnea or shortness of breath (SOB) on exertion. Her peripheral edema decreased to 1+ bilaterally. A transthoracic echocardiography (TTE) was performed, and it showed a moderate concentric LVH. The right ventricle (RV) was moderately enlarged with hypertrophied, and the LV ejection fraction (LVEF) was 60-64% with grade I diastolic dysfunction with elevated left atrial filling pressures. There was severe bilateral atrial enlargements. The PA size was severely enlarged measuring 51 mm and a PDA with a left-to-right shunt. Pulmonary artery systolic pressure (PASP) was 70 mmHg (systemic systolic pressure of 113 mmHg) (Figures 3, 4).

The patient was informed of her diagnosis of a PDA, and a referral to an adult congenital cardiology clinic was sent. However, she was reluctant to travel to another city to be evaluated and refused referral to heart catheterization or outpatient therapy other than a short course with oral furosemide. Upon discharge, the patient was asymptomatic without oxygen supplementation on furosemide 20 mg twice a day and potassium chloride 20 mEq per day. She returned for a follow-up appointment 10 days after the discharge and had stopped therapy due to polyuria. She had gained 2 kg, the lower extremity peripheral edema and functional capacity were unchanged, and she refused therapy or referral again. The patient was readmitted to the hospital 14 months later, with acute hypoxic respiratory failure, acute encephalopathy due to heart failure exacerbation, and acute kidney injury. Her oxygen saturation on room air was 74% on her fingers and 60% on her toes. BP was 86/40 mmHg, and HR was irregular, around 130-150 bpm. The EKG showed atrial fibrillation with a rapid ventricular response. Bedside echocardiogram showed a mildly decreased LV function with an LVEF of 35%, moderate LVH, RVH, and decreased RV systolic function with a PASP of 56 mmHg. The patient suffered emergent electric cardioversion and received guideline-directed medical therapy for heart failure with reduced ejection fraction and atrial fibrillation. She was discharged on apixaban, carvedilol, lisinopril, and torsemide, with an improvement of symptoms, but she needed 2 L/min of supplemental oxygen via nasal cannula.

The patient refused further interventions, such as cardiac catheterization and referral to a specialized center.

## Discussion

Adult congenital heart disease is rare in the elderly population, but in rural areas of the world, specialized testing may be a limiting factor for early diagnosis. PDA is a rare disease in adults, which may present in a wide variety of signs and symptoms. The patient may be asymptomatic but has a continuous, systolic, and diastolic murmur heard at the upper left sternal border, also described as a “machinery murmur” [3]. As seen in this case, this patient was asymptomatic for 84 years, had not seen a provider for 40 years, and only had a diagnosis of “murmur during pregnancy.” The most common presentation in the elderly is heart failure, but pulmonary hypertension, atrial fibrillation, endocarditis, or pneumonia have been described [4]. Chronic left-to-right shunt leads to volume overload and left ventricle remodeling predisposing patients to heart failure. Symptoms will develop depending on the magnitude of shunting and resistance of the PDA (length, diameter, and configuration) [5]. When the pulmonary pressure is elevated, the shunt becomes right-to-left and bypasses the alveolar circulation, and deoxygenated blood enters the systemic circulation downstream from the ductal insertion into the aorta, causing cyanosis in the lower extremities. Accurate assessment of oxygen saturation by oximetry and assessment of cyanosis should be done in the feet and both hands [6].

Diagnosis is confirmed with a TTE, so it is a valuable tool to estimate volume load, systemic to pulmonary flow ratio, left and right ventricular mass and function, and PASP and also helps to identify other associated cardiac pathologies. The cost and availability of TTE have improved worldwide, yet in rural settings, it may still pose a challenge. Furthermore, in individuals with a large PDA with a chronic high left-to-right shunt ratio, EKG may show left atrium dilatation and left ventricle strain [7]. Other imaging modalities, such as CT or magnetic resonance imaging, may also aid in confirming diagnosis [3]. In addition to the standard diagnostic tools, a cardiac catheterization still has an important role in evaluating pulmonary hypertension, vascular resistance, degree of shunting, and even a balloon occlusion test to further stratify the prognosis and risk of DA closure [6-8].

Surgical closure remains the method of choice for the treatment of PDA, including traditional ligation or transcatheter closure [9]. Surgical techniques are influenced by factors, such as the size, shape, calcification, and friability of the PDA [3]. If large and complex forms of PDA are present, it may not be amenable for catheter closure, and surgical closure is warranted [7,10].

The American Heart Association/American College of Cardiology guidelines recommends PDA device closure in patients with signs of volume overload and LV remodeling and pulmonary hypertension if pulmonary vascular resistance is less than one-third of the systemic and/or PASP is less than 50% of the systemic, with a history of infective endocarditis. Pulmonary/systemic shunt ratios of less than 1.5 and pulmonary vascular resistances of greater than 5 Wood units prohibit ductal closure in adults. Once Eisenmenger physiology is established, DA closure is contraindicated due to higher mortality [6].

Supportive pharmacologic treatments (e.g., endothelin receptor antagonists and phosphodiesterase-5 inhibitors) have been shown to improve functional capacities in adult patients with Eisenmenger syndrome, including marked increases in survival advantage for those on PAH therapies versus not on PAH therapies (97% versus 69%; P< 0.01) [11].

The mortality of untreated PDA in adults is 1.8% per year, with 34% of PDA patients dying before the age of 40 and 61% before the age of 60 [3,7,8,12].

## Conclusions

This case report presents the importance of considering PDA as a differential diagnosis in elderly adults. Diagnosing it depends on high clinical awareness since signs and symptoms are nonspecific and overlap with other more prevalent cardiac conditions, including heart failure, pulmonary hypertension, and atrial fibrillation. Due to the complexity of the case, a holistic approach, considering patient preferences and the potential benefits of further interventions, is necessary. This case underscores the significance of diagnosing PDA in the elderly, especially in underserved populations that may suffer from underdiagnosis and present barriers to appropriate therapy.
